# Overexpression of Growth-Related Oncogene-*β* Is Associated with Tumorigenesis, Metastasis, and Poor Prognosis in Ovarian Cancer

**DOI:** 10.1155/2015/387382

**Published:** 2015-04-30

**Authors:** Qing Ye, Xiaolu Zhai, Wei Wang, Shu Zhang, Huijun Zhu, Di Wang, Chenyi Wang

**Affiliations:** ^1^Department of Obstetrics and Gynecology, Affiliated Hospital of Nantong University, Nantong 226001, China; ^2^Department of Oncology, Affiliated Hospital of Nantong University, Nantong 226001, China; ^3^Department of Pathology, Affiliated Hospital of Nantong University, Nantong 226001, China

## Abstract

*Background*. Growth-related oncogene- (GRO-) *β* is a member of the CXC chemokine family, which may mediate various functions, such as attracting neutrophils to sites of inflammation, regulating angiogenesis, and participating in tumorigenesis and progression. However, the expression of GRO-*β* in ovarian cancer and its relationship to the clinical characteristics of this disease remain poorly understood. *Methods*. In this study, immunohistochemical analysis using tissue microarray (TMA) was employed to evaluate the expression of GRO-*β* in ovarian cancer and to contrast expression with normal ovarian epithelial cells and oviduct epithelial cells. Next, we observed the correlation between GRO-*β* expression and clinicopathological features of ovarian cancer as well as patient outcome. *Results*. High GRO-*β* cytoplasmic expression was observed in 55.15% of patients with ovarian cancer, which was related to lymph node or other metastases (*P* < 0.001), ascites (*P* = 0.027), and International Federation of Obstetricians and Gynaecologists (FIGO) stage (*P* = 0.032). Kaplan-Meier survival and Cox regression analysis revealed that high GRO-*β* expression (*P* = 0.002) and high CA19-9 level (*P* = 0.003) were independent prognostic indicators of poor outcome in ovarian cancer. *Conclusions*. Overall, high GRO-*β* expression correlates with poor prognosis and contributes to ovarian cancer tumorigenesis and metastasis.

## 1. Introduction

Ovarian cancer remains the leading cause of death among gynecological tumors. Epithelial ovarian cancer accounts for more than 90% of all malignant ovarian tumors and is the fifth most common cause of cancer-related death among women [1]. According to the initial International Federation of Obstetricians and Gynaecologists (FIGO) stage, the prognosis of ovarian cancer varies; the 5-year survival rate reaches 90% when the disease is limited to the ovary, but it drops to below 50% for cases in which cancer has spread outside the pelvis [2]. Current treatment for advanced ovarian cancer includes debulking and chemotherapy, mainly the combination of paclitaxel and platinum agents, and at least 70% of the patients treated with this combination initially respond to treatment [3]. Despite significant advances in surgical resection and systemic chemotherapies, the long-term outcome remains poor and the 5-year survival is only approximately 30% after the initial diagnosis [4]. The main reason for the poor rate of survival is that there is a lack of early specific symptoms and most of the patients have advanced stage disease (FIGO stages III and IV) at diagnosis. In addition, primary or secondary multidrug resistance also accounts for ovarian carcinoma treatment failure [5]. Therefore, there is an urgent need for novel biomarkers to improve therapeutic methods and extend the survival of ovarian cancer patients.

Chemokines are a superfamily of proinflammatory 70–80 amino acid peptides that attract, activate, and regulate leukocytes by binding to G protein-coupled receptors on the cell surface [6, 7]. In addition to their effect on chemotactic migration of leukocytes, chemokines were shown to play different roles in tumor development through their effect on angiogenesis, hematopoiesis, metastasis, and tumor rejection [8–11]. Chemokines can be classified into three subfamilies, C, CC, or CXC, based on the number and arrangement of conserved cysteine residues [6, 12, 13]. Growth-related oncogene (GRO) is a member of the CXC chemokine family, which is composed of GRO-*α*, GRO-*β*, and GRO-*γ* [14, 15]. Accumulating data suggest that GRO-*α* is involved in tumor development and invasion in various malignancies, such as colorectal cancer [16, 17], prostate cancer [18], and bladder cancer [19]. However, the roles of GRO-*β* in tumors are poorly understood.

In the present study, we investigated GRO-*β* protein expression in a number of ovarian cancer samples by immunohistochemistry using tissue microarray (TMA) sections. Moreover, we assessed the association between GRO-*β* expression and clinicopathological factors to determine its clinicopathological significance in select group of ovarian cancer patients. Finally, we evaluated the prognostic significance of GRO-*β* protein expression levels in ovarian cancer.

## 2. Materials and Methods

### 2.1. Patients and Samples

Formalin-fixed paraffin-embedded malignant ovarian cancer (*n* = 136), borderline adenoma (*n* = 37), benign adenoma (*n* = 33), normal ovarian epithelial tissue (*n* = 20), and oviduct epithelial tissue (*n* = 26) specimens from patients who underwent surgery between 2004 and 2009 were obtained from the Affiliated Hospital of Nantong University. Clinical data (including age, histological type, differentiation, FIGO stage, and follow-up, including 5-year survival and other information) were obtained from the medical records of each patient. Tumor stage was in accordance with FIGO criteria, whereas differentiation and histological type were determined following World Health Organization standards. None of the patients received adjuvant chemotherapy, radiation therapy, or immunotherapy. Survival was calculated from the date of surgery until death or last follow-up. Representative 2.0 mm tissue cores from each patient were used to conduct TMA analysis using a Tissue Microarray System (Quick-Ray, UT06, UNITMA, Seoul, Korea). Ethical approval for this study was obtained from the Human Research Ethics Committee of the Affiliated Hospital of Nantong University.

### 2.2. Immunohistochemistry

For immunohistochemistry (IHC) analysis, the TMA sections were deparaffinized in 100% xylene and rehydrated in graded ethanol solutions. The sections were then boiled under pressure in citrate buffer (pH 6.0) for 5 minutes for antigen retrieval. TMA sections were incubated overnight with a primary anti-GRO-*β* antibody (Catalog 500-P104, PeproTech, Rocky Hill, NJ, USA) diluted 1 : 400 in TBS containing 1% bovine serum albumin. After washing, sections were incubated with anti-rabbit horseradish peroxidase-conjugated antibody (Santa Cruz Biotechnology, Santa Cruz, CA, USA). GRO-*β* immunostaining was evaluated independently by two trained pathologists who were blinded to the clinical background of the cases. Positivity of cell staining was recorded as a percentage (0–100%).

The cutoff point for the GRO-*β* expression score that was statistically significant in terms of overall survival (OS) was determined using the X-tile software program (Rimm Lab, Yale University, New Haven, CT, USA), as described elsewhere [20]. The degree of staining was quantified using a two-level grading system, and staining scores were defined as follows: for GRO-*β*, 0–75 was regarded as low expression while 76–100 was regarded as high expression.

### 2.3. Statistical Analysis

Relationships between clinicopathological factors and GRO-*β* expression were examined using *χ* ^2^ tests. For the TMA slides, the following clinical data were assessed: age, histological type, differentiation, FIGO stage, and other clinicopathological information. Univariate and multivariate analyses were evaluated using Cox proportional hazards regression models. Survival curves were calculated using the Kaplan-Meier method and compared with the log-rank test. For all statistical analyses, *P* values less than 0.05 were regarded as statistically significant. Statistical analyses were carried out using STATA V.9.0 software (Stata Corporation, College Station, TX, USA) and SPSS V.20.0 software (IBM Corporation, Armonk, NY, USA). All statistical tests were two-sided.

## 3. Results

### 3.1. Clinical Features of Ovarian Cancer

The age of patients ranged from 24 to 80 years, with an average age of 55.26 years. There were 89 serous adenomas, 13 endometrioid tumors, nine clear cell tumors, six transitional cell carcinomas, seven mucinous cystadenocarcinomas, and 12 mixed tumors. All cases were stratified according to FIGO (I-II, 80 cases; III, 53 cases; and IV, three cases). Detailed clinicopathological data are shown in Table 1.

### 3.2. Expression of GRO-*β* in Ovarian Cancer by IHC Analysis

**Figure 1:**
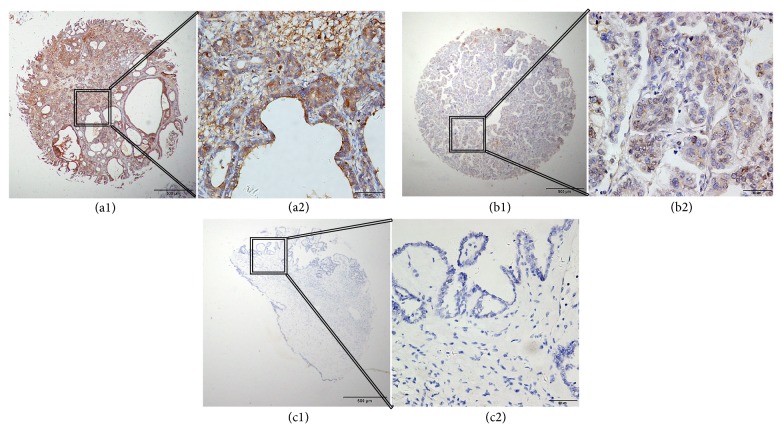
Representative immunohistochemistry (IHC) images showing expression of GRO-*β* in tissue microarray sections of ovarian cancer. (a1) and (a2) show strong positive staining in the cytoplasm. (b1) and (b2) show weakly positive staining in the cytoplasm. (c1) and (c2) show a negative IHC reaction in benign ovarian tumor. Original magnification was ×40 for (a1), (b1), and (c1) and ×400 for (a2), (b2), and (c2).

We performed IHC analysis to examine GRO-*β* expression in ovarian cancer. Positive staining was localized mainly in the cytoplasm of ovarian cancer cells. High GRO-*β* cytoplasmic expression was detected in 75 (55.15%) of 136 cases of malignant ovarian cancer, four (10.81%) of 37 cases of borderline adenomas, zero (0.00%) of 33 cases of benign adenomas, zero (0.00%) of 20 cases of normal ovarian epithelial cells, and one (3.85%) of 26 cases of oviduct epithelial cells. Furthermore, *χ* ^2^ analysis also revealed that high GRO-*β* expression was significantly associated with ovarian tumor progression (*χ* ^2^ = 75.847, *P* < 0.001). Typical IHC staining patterns for GRO-*β* in ovarian cancer are shown in Figure 1.

### 3.3. Association between GRO-*β* Expression and Clinicopathological Parameters

The relationship between high GRO-*β* expression and clinicopathological features of 136 cases of ovarian cancer is shown in Table 1. High GRO-*β* cytoplasmic expression was related to FIGO stage (*P* = 0.032), ascites (*P* = 0.027), and lymph node or other metastases (*P* < 0.001). In contrast, no statistically significant correlation was found between GRO-*β* expression and other clinical parameters, including age, histological type, or differentiation.

### 3.4. Survival Analysis

**Figure 2:**
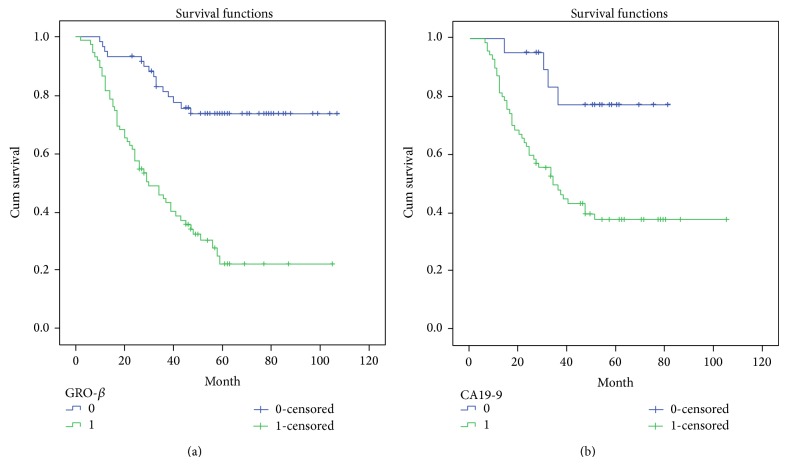
Analysis of survival of ovarian cancer patients by Kaplan-Meier survival curve. (a) Survival curves calculated by growth-related oncogene- (GRO-) *β* expression. GRO-*β* = 1 is the high expression group (green line), while GRO-*β* = 0 is the low and no expression group (blue line). (b) Survival curves based on CA19-9. CA19-9 = 1 represents the group with high serum CA19-9 levels (green line), while CA19-9 = 0 represents the group with low serum CA19-9 levels (blue line).

Several known predictive factors of poor outcome in ovarian cancer were assessed to confirm that our patient cohort was representative of those with ovarian cancer (Table 2). As expected, GRO-*β* protein overexpression (*P* < 0.001) was significantly associated with 5-year survival by Cox regression univariate analysis. In addition, other prognostic factors, such as age (*P* = 0.028), histological type (*P* = 0.017), FIGO stage (*P* < 0.001), CA19-9 level (*P* = 0.007), CA153 level (*P* = 0.021), and lymph node and other metastases (*P* < 0.001), were also statistically significant. All these factors were included in the multivariate analysis. High GRO-*β* expression (*P* = 0.002) and high CA19-9 level (*P* = 0.003) were identified as independent predictive factors of poor outcome in ovarian cancer. Kaplan-Meier survival curves demonstrated that patients with high GRO-*β* expression and high CA19-9 level had a significantly shorter survival time (Figure 2).

## 4. Discussion

The majority of ovarian cancer patients are not diagnosed until the disease is in an advanced stage because of diffuse symptoms. Therefore, to improve the prognosis of patients with this pernicious disease, identification of targets for early detection of ovarian cancer is critical. Numerous efforts have been made to evaluate biomarkers that screen the population cohort at risk but so far without substantial success. Even the most common tumor marker, CA125, is not reliable because of low sensitivity and specificity in patients with early-stage ovarian cancer [21]. Thus, more novel tumor biomarkers with high sensitivity and reasonable specificity for ovarian cancer are urgently needed.

In cancer, chemokines and their receptors play a crucial role in the trafficking of cells in and out of the tumor microenvironment, thereby modulating the behavior of the tumor. In addition to their roles in the immune system, CXC chemokines and their receptors are also involved in tumor initiation, progression, and metastasis [22, 23]. Experimental evidence indicates that GRO-*β*, also known as the chemokine CXCL2, may mediate varied functions, such as attracting neutrophils to sites of inflammation, regulating angiogenesis, and modulating neurotransmitter release [24–26]. GRO-*β* is a member of the CXC chemokine family, which includes the related ligands GRO-*α*, GRO-*γ*, ENA78, neutrophil-activating peptide- (NAP-) 2, and interleukin-8 (IL-8), and it has biological activities related to specific binding to the CXCR2 receptor [27, 28]. Through binding to its receptor, CXCR2, GRO-*β* forms an autocrine loop that activates the Ras-Erk1/2 signaling pathway, which is important for cell proliferation [26, 29]. This pathway in turn enhances the transcription and expression of early growth response protein- (EGR-) 1, a transcription factor that regulates the expression of downstream factors related to cell growth and cell cycle regulation, thereby promoting tumor progression [30, 31].

The roles of GRO-*β* in tumor formation and development have been previously investigated. Recent studies have reported that GRO-*β* is associated with tumor development and invasion. For instance, Wang et al. reported that GRO-*β* is highly expressed in esophageal squamous cell carcinoma (ESCC) tissues and cell lines [29]. Dong et al. found that the serum GRO-*β* levels are much higher in ESCC patients than in healthy controls. Finally, Doll et al. reported significantly increased expression of GRO-*β* in colon carcinoma compared with normal tissue [32]. These studies indicate that GRO-*β* plays a crucial role in the development and metastatic processes in several malignancies. In line with these studies, we found that GRO-*β* was significantly increased in ovarian cancer specimen compared with benign ovarian tumors.

To further investigate the biological roles of GRO-*β* in ovarian cancer, we analyzed the correlation between GRO-*β* expression and prognosis in ovarian cancer patients. In the present investigation, GRO-*β* expression in ovarian cancer tissues was evaluated using IHC, and results showed that 55.15% of cases exhibited high GRO-*β* cytoplasmic expression. Furthermore, we found that strong GRO-*β* expression in ovarian cancer was significantly correlated with FIGO stage, ascites, and lymph node and other metastases. Our data clearly showed that high cytoplasmic expression of GRO-*β* was associated with significantly poor survival. Multivariate analyses revealed that GRO-*β* expression was regarded as an independent prognostic factor for ovarian cancer patient. Aside from high GRO-*β* expression, high CA19-9 level is considered an independent factor for poor prognosis in ovarian cancer.

In conclusion, this is the first report of the differential expression of GRO-*β* in ovarian cancer, and it indicates that GRO-*β* may constitute a novel prognostic marker for ovarian cancer. Our findings demonstrated high expression of GRO-*β* in ovarian cancer specimens and demonstrated that this high expression was associated with poor prognosis. Our results provide the basis for future directions in cancer therapy using GRO-*β* protein as a potential molecular target. However, further studies are required to elucidate the signaling pathways and mechanisms underlying GRO-*β* in the development and metastatic process of ovarian cancer and to clarify whether GRO-*β* can be used as a novel therapeutic target.

## Figures and Tables

**Table 1:** Relationship between the expression of GRO-*β* and clinicopathological characteristics in ovarian cancer.

Characteristic	*n*	Low or no expression	High expression	Pearson *χ* ^2^	*P* value
Total	**136**	**61**	**75**		
Age				2.051	0.152
≤60	94	46 (48.94)	48 (51.06)		
>60	42	15 (35.71)	27 (64.29)		
Histological type				1.176	0.555
Serous	89	37 (41.57)	52 (58.43)		
Endometrioid	13	7 (53.85)	6 (46.15)		
Others	34	17 (50.00)	17 (50.00)		
Differentiation				1.083	0.298
Low	28	15 (53.57)	13 (46.43)		
High	108	46 (42.59)	62 (57.41)		
FIGO stage				4.593	0.032^∗^
I~II	80	42 (52.50)	38 (47.50)		
III~IV	56	19 (33.93)	37 (66.07)		
CEA level				1.636	0.201
≤5	80	34 (42.50)	46 (57.50)		
>5	13	8 (61.54)	5 (38.46)		
Unknown	43	19	24		
CA19-9 level				0.053	0.817
≤37	70	32 (45.71)	38 (54.29)		
>37	21	9 (42.86)	12 (57.14)		
Unknown	45	20	25		
CA125 level				0.853	0.355
≤35	12	4 (33.33)	8 (66.67)		
>35	82	39 (47.56)	43 (52.44)		
Unknown	42	18	24		
CA153 level				1.457	0.227
≤31	38	20 (52.63)	18 (47.37)		
>31	48	19 (39.58)	29 (60.42)		
Unknown	50	22	28		
SF level				2.686	0.101
≤204	59	30 (50.85)	29 (49.15)		
>204	28	9 (32.14)	19 (67.86)		
Unknown	49	22	27		
Ascites				4.916	0.027^∗^
No	48	26 (54.17)	22 (45.83)		
Yes	42	13 (30.95)	29 (69.05)		
Unknown	46	22	24		
Lymph node and other metastases				26.097	<0.001^∗^
No	60	37 (61.67)	23 (38.33)		
Yes	76	24 (31.58)	52 (68.42)		

^∗^ *P* < 0.05.

Others: clear cell, nine cases; mucinous, seven cases; transitional cell, six cases; mixed, 12 cases.

**Table 2:** Univariate and multivariate analysis of prognostic factors for 5-year survival in ovarian cancer.

	Univariate analysis				Multivariate analysis			
	HR	*P* > |*z*|	95% CI		HR	*P* > |*z*|	95% CI	
GRO-*β* expression								
High versus low	4.450	<0.001^∗^	2.495	7.937	3.789	0.002^∗^	1.659	8.655
Age (years)								
≤60 versus >60	1.733	0.028^∗^	1.060	2.832	1.693	0.163	0.807	3.554
Histological type								
Serous versus endometrioid versus others	0.768	0.017^∗^	0.618	0.954	0.846	0.191	0.660	1.086
Differentiation								
Low versus High	0.496	0.117	0.206	1.192				
Ascites								
No versus Yes	1.659	0.079	0.942	2.919				
Lymph node and other metastases								
No versus Yes	3.425	<0.001^∗^	1.966	5.965	0.737	0.600	0.236	2.301
FIGO stage								
I~II versus III versus IV	1.962	<0.001^∗^	1.511	2.547	3.900	0.119	0.703	3.616
CEA level								
≤5 versus >5	0.823	0.657	0.349	1.941				
CA199 level								
≤37 versus >37	0.241	0.007^∗^	0.086	0.675	0.184	0.003^∗^	0.060	0.568
CA125 level								
≤35 versus >35	1.826	0.249	0.655	5.089				
CA153 level								
≤31 versus >31	2.130	0.021^∗^	1.122	4.045	1.146	0.755	0.487	2.695
SF level								
≤204 versus >204	1.476	0.213	0.799	2.725				

^∗^ *P* < 0.05.

Others: clear cell, nine cases; mucinous, seven cases; transitional cell, six cases; mixed, 12 cases.
